# “The clock is ticking”: high prevalence of tick-borne zoonoses in healthy adults with varying tick-bite exposure in Northwestern Italy

**DOI:** 10.1186/s12879-026-14094-9

**Published:** 2026-07-28

**Authors:** Rachele Vada, Stefania Zanet, Ezio Ferroglio, Anna Trisciuoglio, Amir Reza Varzandi, Andrea Pepe, Enrico Garro, Laura Ponzetta, Giovanni Di Perri, Andrea Calcagno

**Affiliations:** 1 Department of Veterinary Sciences, University of Turin, Grugliasco, Italy; 2 Unit of Infectious Diseases, Department of Medical Sciences, University of Turin, Torino, Italy; 3 Department of Translational Medicine, University of Eastern Piedmont, Novara, Italy; 4 SCDO Malattie Infettive, AOU Maggiore della Carità, Novara, Italy

**Keywords:** Acarological risk, Risk factors, Babesiosis, Lyme disease, Mediterranean spotted fever, Human granulocytic anaplasmosis

## Abstract

Tick-borne zoonoses are among the main vector-borne and emerging diseases in Europe. Individuals with chronic high-frequency tick exposure represent sentinel populations for pathogen circulation. In this work, we report the biomolecular prevalence (PCR) and seroprevalence of *Babesia divergens*, *B. microti*, *Borrelia burgdorferi* s.l., Spotted Fever Group (SFG) *Rickettsiae*, Anaplasmataceae and Tick-borne Encephalitis Virus in a cohort of 144 healthy adults from Northwestern Italy, with varying tick-bite exposure intensity ranging from occasional to > 10 bites/year. Participants were exposed to tick bites through engagement with natural environments, with 65% reporting at least one tick bite per year. Time spent outdoors and checking the body for ticks upon returning home correlated significantly with reported tick-bite frequency. Across all pathogens, biomolecular prevalence was 19%, while seroprevalence was 44%. We detected the highest prevalence for *B. divergens* (22.2% serology) and *B. microti* (13.9% serology), followed by SFG *Rickettsiae* (9.3% PCR, 14.3% serology), *A. phagocytophilum* (6.9% PCR, 12.5% serology), *B. burgdorferi* s.l. (0% PCR, 3.5% serology), with no TBEV infections detected. Despite high pathogen exposure, all participants were asymptomatic at sampling, mirroring patterns observed in wildlife host populations where high infection prevalence coexist with subclinical or asymptomatic infections. These findings are consistent with published data from comparable high-exposure cohorts using similar diagnostic methodologies and emphasize the importance of exposure stratification in tick-borne disease epidemiology. This data is relevant to raise awareness in the general public, to inform medical practitioners, and for epidemiological and public health surveillance actions and disease control.

**Supplementary Information:**

The online version contains supplementary material available at https://doi.org/10.1186/s12879-026-14094-9.

## Introduction

With the increasing abundance and expanding geographic range of their vectors, tick-borne zoonoses (TBZ) have become some of the most significant vector-borne diseases in Europe [1–3]. They encompass a wide range of causative agents, from viruses to protozoa. Historically, research has primarily focused on Lyme borreliosis and tick-borne encephalitis (TBE) due to their high prevalence and public health impact [4]. Simultaneously, the emergence and re-emergence of new tick-borne pathogens in several countries have been increasingly recognized [4–6]. Human babesiosis has been now reported across most of Europe [7]. In addition to *Babesia divergens*, which is the primary aetiological agent of human babesiosis in Europe, an increasing number of cases have also been attributed to other species, including *B. venatorum* and *B. microti* [8]. Although the overall public health burden of TBZ remains largely unquantified, Lyme disease burden was estimated in tens of millions of euros in several European countries [9]. An analysis of hospital discharge records from 2000 to 2012 in Northwestern Italy identified a combined healthcare cost associated with tick-borne diseases of approximately €650,000 [10]. In wildlife epidemiology, high pathogen prevalence frequently coexists with minimal clinical disease in populations with chronic pathogen exposure. For instance, *Anaplasma phagocytophilum* maintains prevalence rates of 40–60% in alpine ungulates (i.e. chamois, *Rupicapra rupicapra*) with sporadic clinical manifestations [11]. Similarly, piroplasms of the genera *Bebesia* and *Theileria* circulate at high levels in European wild cervid and bovid populations without corresponding disease burden (12–14]. *Babesia microti* exhibits prevalence exceeding 40% in rodent reservoir hosts with minimal pathological impact [15]. This dissociation between infection and disease reflects complex host-pathogen adaptations involving immune accommodation, subclinical carrier states, pathogen strain variability, and ecological transmission dynamics.

Humans with chronic high-frequency tick exposure may exhibit analogous epidemiological patterns. Unlike the general population with sporadic tick encounters, individuals spending extensive time in tick-endemic habitats experience repeated pathogen exposure, potentially leading to immunological adaptation, transient subclinical infections, or serological responses without active disease (16–19]. Understanding pathogen circulation in these sentinel populations provides critical insights into enzootic transmission cycles and emerging zoonotic risks at the wildlife-human interface, while also informing interpretation of seroprevalence data in the context of clinical risk assessment. The increasing popularity of outdoor activities [20] coupled with the expansion of areas suitable for ticks’ presence [21] and their extended season of activity [22], is increasing the risk of tick bites for people who spend time in natural settings. This heightened risk affects not only those engaged in occupational activities but also individuals participating in leisure activities including hunting [23], and outdoor sports, from hiking [24, 25] to dog walking [26]. In general, the level of exposure may vary based on several behavioural factors, such as type and frequency of outdoor activity, choice of clothing, use of protective measures and type of habitat frequented [27].

For six of the most common TBZ pathogens detected in ticks in Northern Italy and Europe [28, 29], namely *Babesia divergens*, *B. microti* (including *B. microti*-like), *Borrelia burgdorferi* s.l., spotted fever group (SFG) rickettsiae, members of the Anaplasmataceae family, and tick-borne encephalitis virus (TBEV), we aimed to determine seroprevalence and, for bacterial pathogens, the prevalence of PCR-confirmed active infection in a cohort of healthy adults with varying frequencies of tick exposure and levels of engagement in natural habitats.

## Materials and methods

### Human ethics and consent to participate

The project received approval from the Ethics Committee of the University of Turin (Prot. n. 0346675, 26/06/2023). Written informed consent was obtained from all participants, and all data were anonymized. Good Clinical Practice recommendations and the principles outlined in the Declaration of Helsinki were followed.

### Sample collection

This study employed a cross-sectional design targeting healthy adults residing in Piedmont Region (Northwestern Italy) with varying degrees of tick-bite exposure through natural environment engagement. Participants were recruited voluntarily during summer-autumn 2023 through associations linked to the University of Turin. Recruitment focused on individuals at elevated baseline risk of exposure, with documented frequent natural habitat use (high-risk) through various activities: wildlife research and field biology, forestry management, hunting, and recreational outdoor activities. Participants were subsequently characterized by quantitative and qualitative exposure variables including self-reported annual tick-bite frequency, types of habitats visited, duration and frequency of outdoor activities, residential exposure (living in peri-urban/rural settings with direct habitat access), and adoption of protective behaviors. Within this population, participants were classified according to relative exposure intensity into a higher-exposure group (professional exposure and frequent off-trail outdoor activities such as hunting) and a lower-exposure group (occasional outdoor activity). This approach allows assessment of pathogen circulation patterns within a high-risk population.

During sampling procedures, blood was collected from each participant in two tubes: one tube without anticoagulant (6 ml) for serological analysis, and one EDTA tube (6 ml) for biomolecular detection of pathogens. Concurrently, participants completed a structured questionnaire collecting demographic information, detailed exposure history, tick-bite history, protective behavior practices, and relevant medical history including previous tick-borne disease diagnoses and treatments (Supplementary Table 1).

### Serological analysis

Sera was separated by centrifugation and stored in individual vials at − 20 °C until analysis. Human IgG antibody detection was performed using commercially available kits whose use was supported by specific scientific literature (Supplementary Table 2). We used IFAT kits for detecting IgG against *B. divergens*, *B. microti* and *A. phagocytophilum*, and ELISA kits for TBEV, SFG *Rickettsiae* and *B. burgdorferi* s.l. Due to the absence of a commercially available assay targeting all SFG *Rickettsiae*, we used an ELISA assay specific for *R. conorii*, species endemic in the study area and for which the manufacturer reported a high degree of cross-reactivity with other SFG species (from the kit manual, Supplementary Table 2). Results were thus referred to as SFG *Rickettsiae*. For similar reasons, we selected an assay targeting the Anaplasmataceae species most relevant for zoonotic risk, namely *A. phagocytophilum*. However, cross-reactivity with *(A) platys* was reported by the producer. According to the manufacturer, the TBE ELISA assay may exhibit cross-reactivity with other Flaviviruses (which were excluded through viral seroneautralization [30]), while the *B.* *burgdorferi* assay may cross-react with *Leptospira* spp., *Treponema pallidum*, and rheumatoid factor, which were excluded by double testing with Immunoblotting. Although no cross-reactivity has been reported for the *B. microti* assay, the *B. divergens* IFAT may cross-react with *Plasmodium* spp. and other *Babesia* species. To the best of our knowledge, for *B. venatorum* there was no commercial kit available at the time of the study (also reported by Wilhelmsson and colleagues [31]). All samples were tested following the instructions provided by the respective manufacturers. Due to variability in our kits, we incorporated only the qualitative (positive-negative) outcomes into our analysis. For IFAT, titres ≥ 1:64 were considered as positive for the purposes of this study. TBE ELISA kit did not allow to differentiate vaccinated individuals which were identified through the survey.

### Biomolecular analysis

Molecular assays were additionally performed for bacterial pathogens to assess active infection. EDTA tubes were stored at -20 °C until further analysis. DNA extraction was performed from 200 µl of blood, with the PureLink Genomic DNA Mini Kit (Invitrogen, CA, USA), according to the manufacturer’s instructions. The quality and quantity of extracted DNA were evaluated with Nanodrop One (ThermoFisher, MA, USA).

We performed amplicon-based pathogen screening through end-point PCR (Supplementary Table 3). Results and species were confirmed by sequencing of positive amplicons (Macrogen Europe, Amsterdam, The Netherlands) and comparison with sequences available in GenBank^®^. All standards precautions were taken to minimize the risk of cross-contamination or laboratory environment contamination, and water template (negative) controls as well as positive control samples were included in all analytical procedures. Positive controls were either DNA extracted from cultivated pathogen or positive samples previously confirmed by amplicon sequencing. For end point PCR amplicons were analysed by agarose gel electrophoresis (2%) and visualized by staining with Gel Red Nucleic Acid Gel Stain (VWR International Milano, Italy). Positive samples were considered based on amplicon bands visualization and height.

### Statistical analysis

We conducted exploratory and inferential analyses to characterize exposure patterns and their relationships with protective behaviours, tick-bite frequency, and pathogen detection. Associations among exposure-related variables were first assessed using chi-squared tests for binary outcomes to identify correlated variables and reduce redundancy in subsequent analyses. Correlations between exposure frequency, protective measures, and tick-bite frequency—categorized into five ordinal levels—were evaluated using Spearman’s rank correlation coefficient. Finally, associations between exposure stratification groups and molecular and serological outcomes were assessed using chi-squared tests, considering all detected pathogens jointly. Analysis was performed in R Studio [32].

## Results

### Exposure characteristics of study cohort

**Fig. 1:**
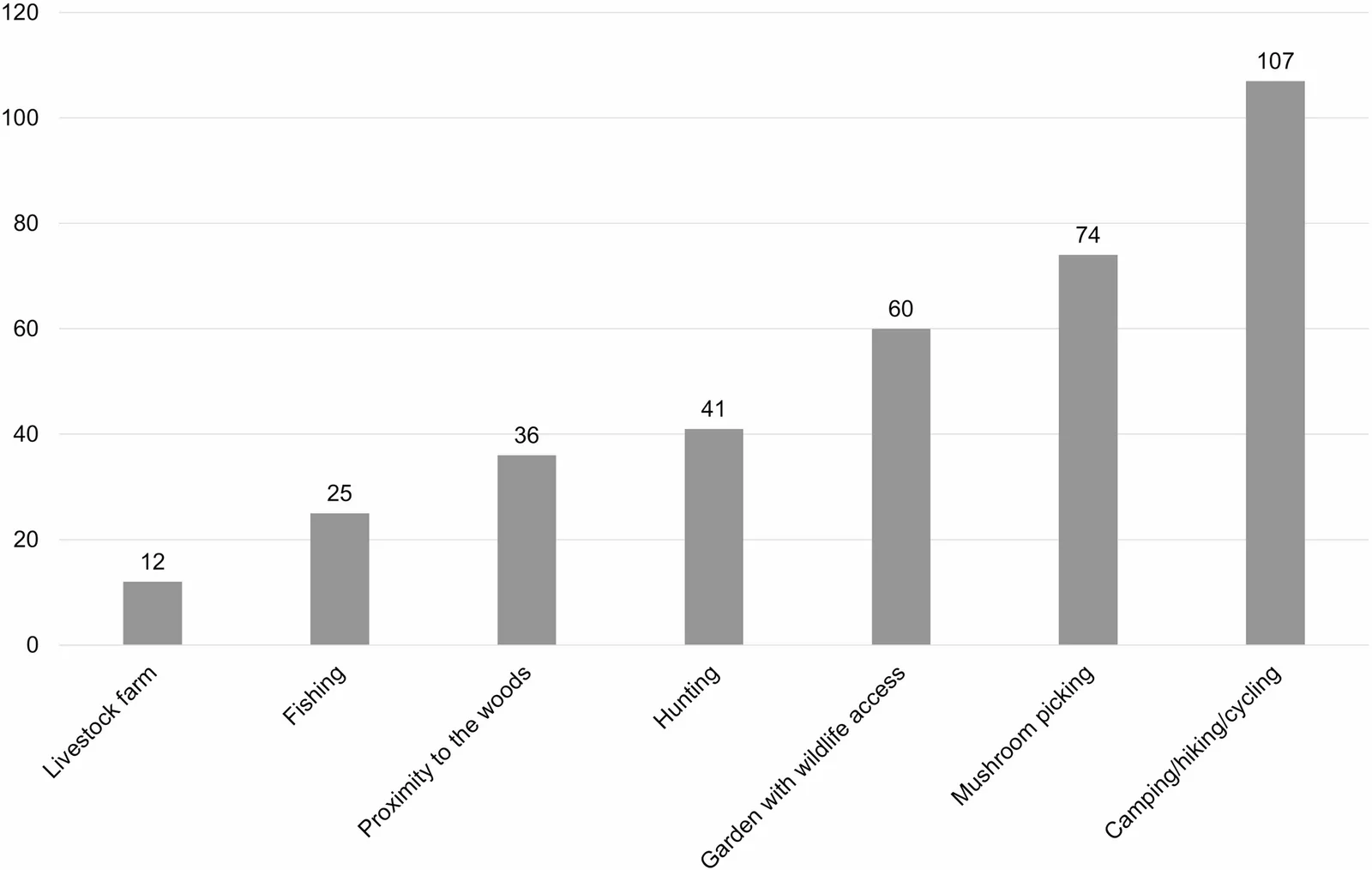
Frequency distribution (as sum of individuals) of exposure behaviours/housing conditions to tick bite

A total of 144 healthy adults were enrolled (male-to-female ratio 1.9:1; median age 28 years). The cohort exhibited a wide range of tick-bite exposure intensities based on self-reported annual tick-bite frequency: 34% (49 individuals) reported no tick bites in the previous year, 24% (34 individuals) reported 1 or fewer, 27% (38 individuals) reported 1–5 bites, 4% (6 individuals) reported 5–10 bites, and 8% (11 individuals) reported more than 10 tick bites annually. Cumulatively, 65% of participants reported at least one tick bite per year, with 19% reporting ≥ 5 bites annually. We detected a significant correlation of the frequency of tick bite and stratification group (Spearman correlation rho = 0.52, *p* < 0.001). Residential exposure, defined as living in peri-urban or rural settings with direct access to natural habitats, was reported by 54% of participants (Fig. 1). The majority (91%) engaged in at least one regular outdoor recreational activity, with hiking/trekking (45%), hunting (25%), and wildlife observation/field research (30%) being most common. Professional outdoor work was reported by 48% of participants, including forestry management, wildlife biology, and environmental monitoring roles. When asked where ticks were most frequently encountered, participants identified woodlands (72%) and meadows (61%) as primary tick-exposure sites, consistent with known *Ixodes ricinus* habitat preferences and questing behavior patterns. Exposure patterns were associated with the stratification group (p value of Chi-squared test > 0.05), therefore only the latter has been used in the subsequent analysis. Frequency of the activity also correlated with the stratification group (Spearman correlation rho = 0.62, *p* > 0.001).

**Fig. 2:**
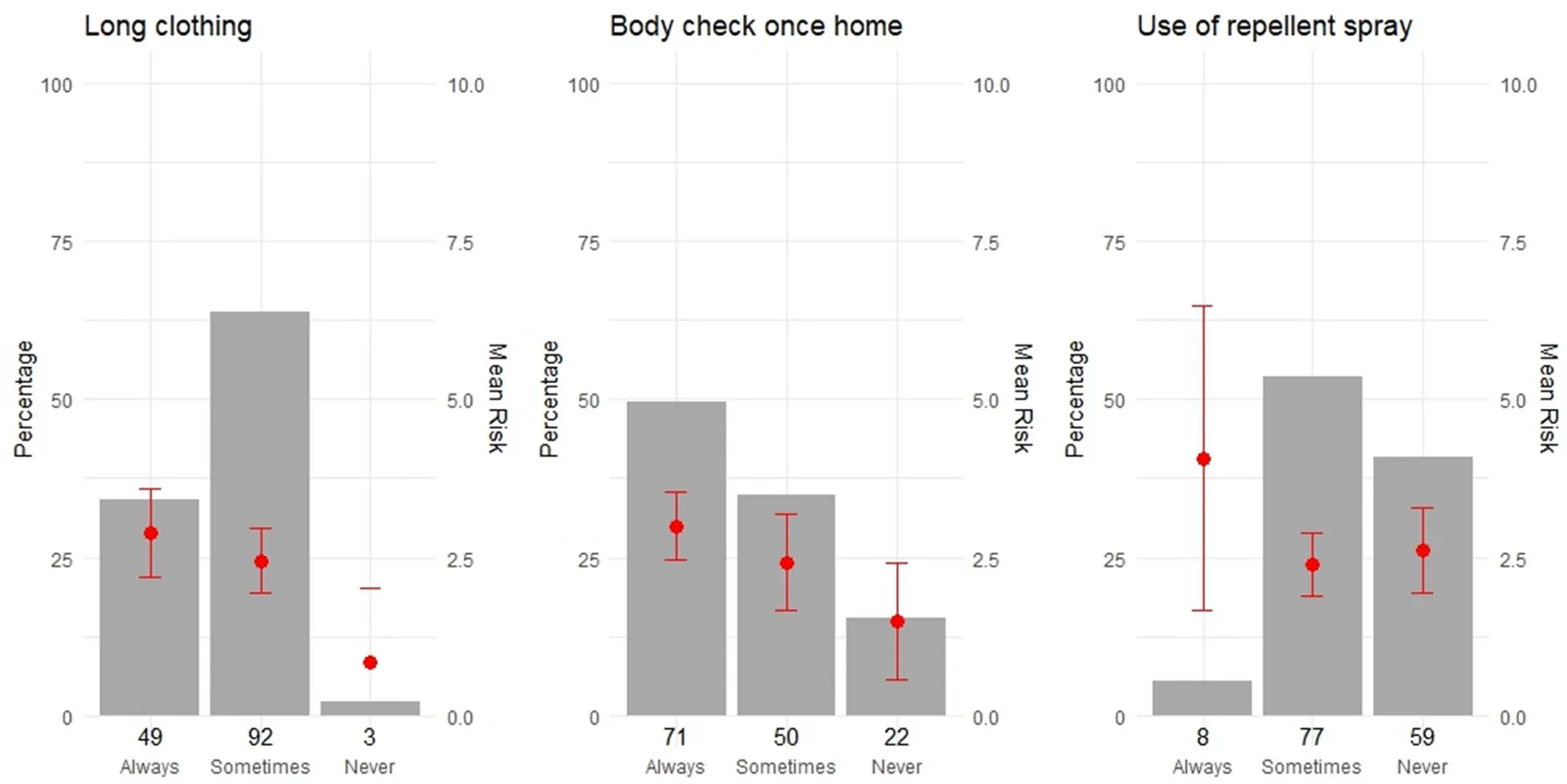
Frequency distribution (in percentage, illustrated by grey bars and referring to left y axis) of the use for the three protective behaviours investigated in the survey. For comparison, we introduced the mean risk for each category of frequency (red whisked dots, referring to right axis)

Among the different methods used to prevent tick bites and the transmission of tick-borne pathogens, the practice of conducting thorough body checks upon returning home was the most commonly adopted strategy (Fig. 2). Participants who experienced longer exposure to tick bites (measured by the hours per day and days per week spent engaging in outdoor activities) were more likely to consistently wear long clothing (Spearman correlation rho = 0.18, *p* = 0.03) and check for ticks once they returned home (Spearman correlation rho = 0.34, *p* < 0.001).

After a tick bite, only 26 participants reported symptoms: 4 people reported severe symptoms possibly related to TBPs (joint pain, fever, loss of appetite, headache) and 22 reported mild symptoms, likely directly related to tick bite itself rather than to tick-borne infections (redness, swelling, itching, erythema at the biting site). One individual reported medical history of chronic Lyme disease from tick bite, although he was not showing symptoms at the moment of sampling.

### Biomolecular and serological analysis

**Fig. 3:**
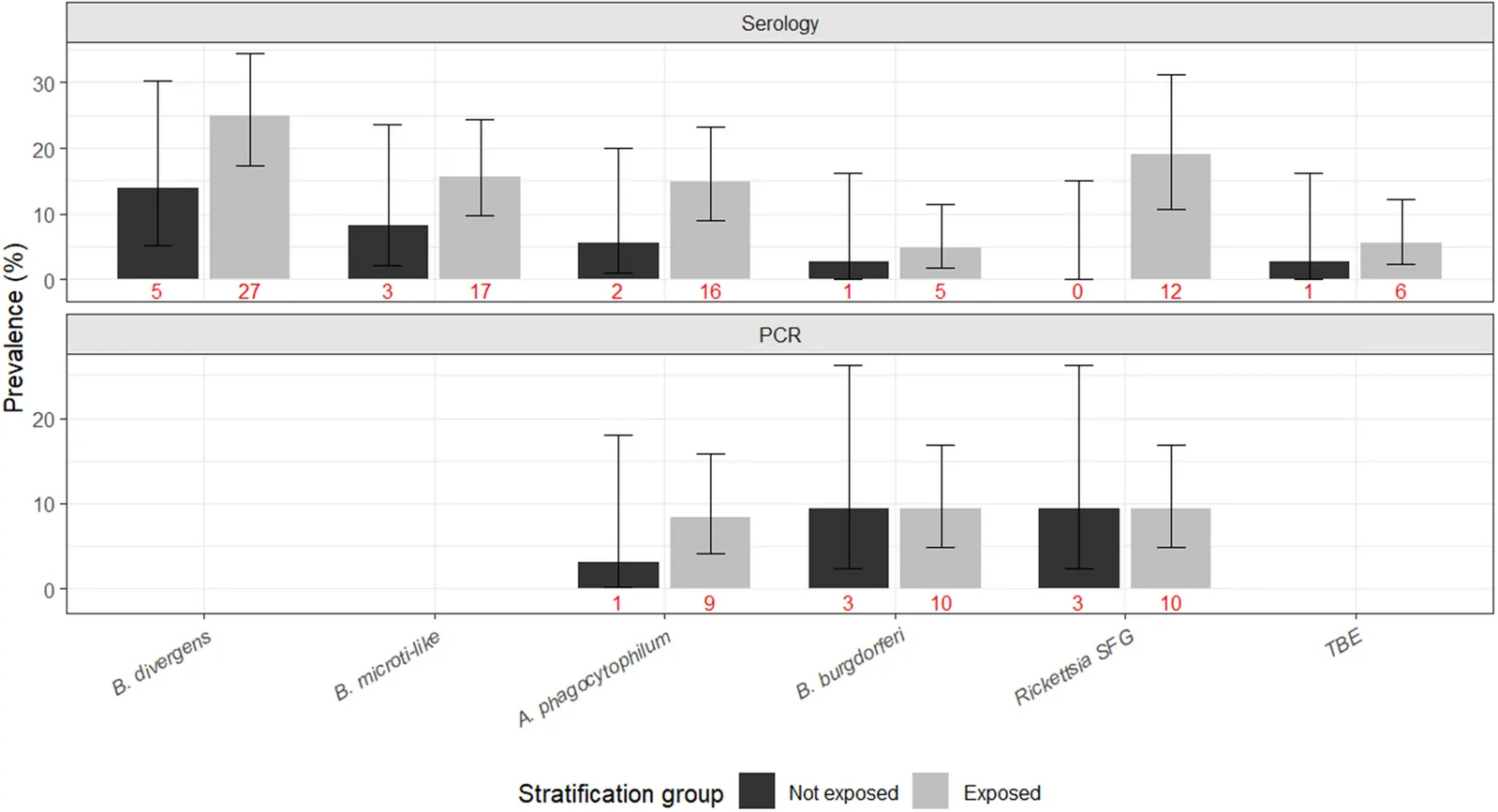
Seroprevalence and PCR of targeted pathogens, with 95% C.I., comparing the two stratification groups; the raw number of positive individuals is below the column

Across all pathogens, the overall biomolecular prevalence was 19%, while seroprevalence was 44%. The highest prevalence and seroprevalence was detected for *Babesia* spp. (*B. divergens* serology 22.2%, 95% C.I. 15.4%-29% [32 positives]; *B. microti*-like serology 13.9%, 95% C.I. 8.2%-19.5% [20 positives]), followed by SFG *Rickettsiae* (PCR 9.03%, 95% C.I. 4.35%-13.71% [13 positives] and serology 14.3%, 95% C.I. 7.1–21.5% [12 positives out of 90 tested]) and *A. phagocytophilum* (PCR 6.9%, 95% C.I. 2.8%-11.1% [10 positives] and serology 12.5%, 95% C.I. 7.1%-17.9% [18 positives]). No positive results for obtained for *Borrelia burgdorferi* s.l. by PCR, while seroprevalence was 3.5%, 95% C.I. 0.5–6.5% [6 positives]. All four individuals who tested positive by TBEV ELISA had been previously vaccinated against TBEV; therefore, no evidence of natural exposure to the virus was detected. The same trends were mirrored in each group (Fig. 3, Supplementary Table 4 for prevalence with 95% C.I. values). All amplicons obtained from Anaplasmataceae-specific PCR assays were identified as *A. phagocytophilum* (GenBank: KP245908.1; query cover ranging from 93 to 100% and a percentage of identity from 95.49 to 100%; accession numbers from PQ657427 to PQ657433). Similarly, all amplicons generated by SFG Rickettsia-specific PCR were identified as *Rickettsia monacensis* (GenBank: LN794217.1; query cover of 100% and a percentage of identity from 97% to 100%; accession numbers from PQ684452 to PQ684460).

**Fig. 4:**
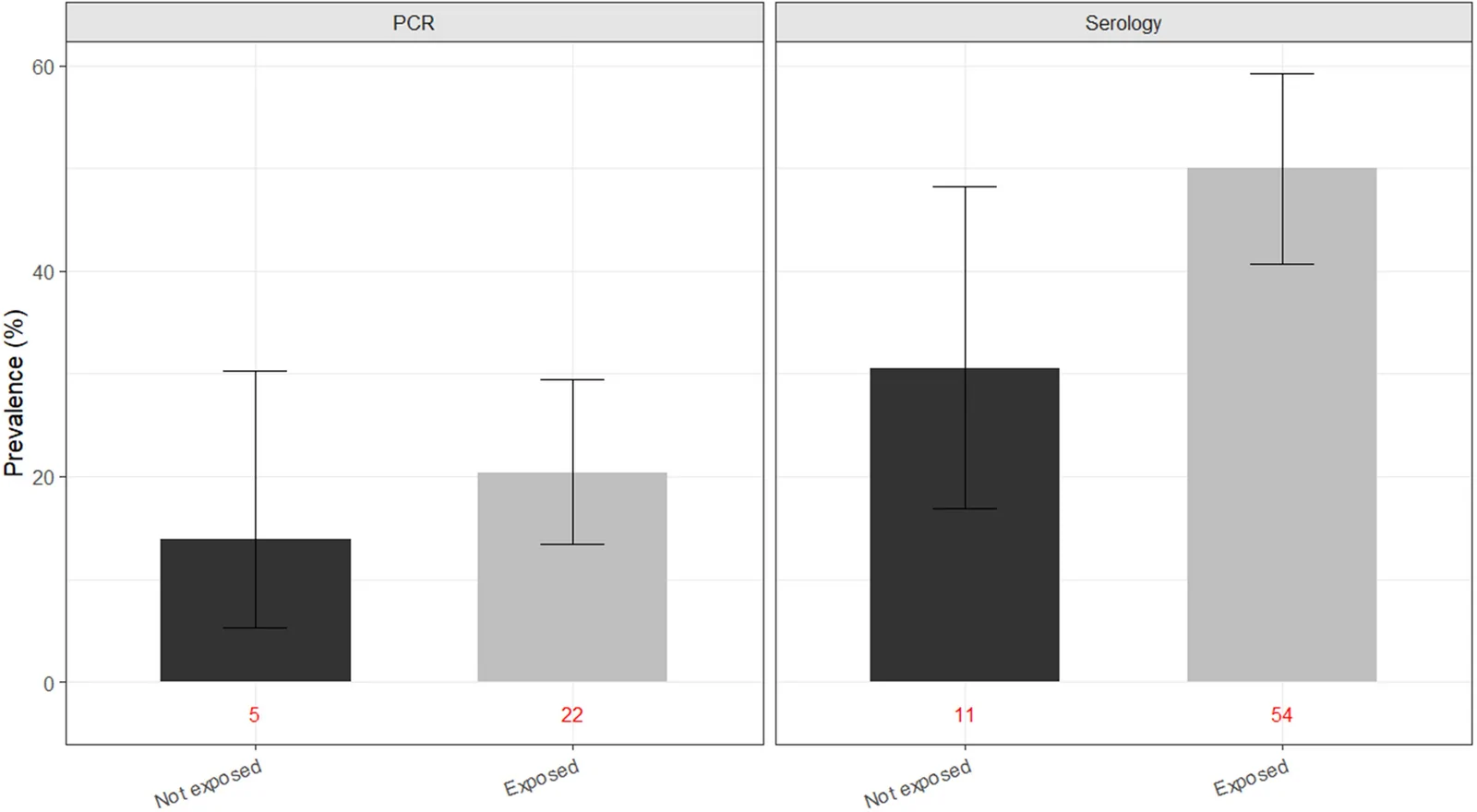
Seroprevalence and PCR considering all targeted pathogens, with 95% C.I., comparing the two stratification groups

The overall seroprevalence was higher in the stratification group with higher exposure (Chi squared = 5.25, *p* = 0.02 for serology, Fig. 4).

## Discussion

This study characterizes tick-borne pathogen prevalence and seroprevalence in a cohort of healthy adults with high-frequency tick exposure in Northwestern Italy, with an overall biomolecular prevalence (19%) and seroprevalence (44%) across multiple pathogens occurring in the absence of clinical symptoms at the time of sampling.

Nearly 90% of all study participants reported engaging in recreational outdoor activities, with varying degrees of exposure to tick bites. In our study, weekly frequency of activity in the natural environment is the main factor positively affecting the risk of tick bite. This concept was already presented by a study in Germany [26]. Reported annual tick-bite frequencies in our cohort (65% ≥1 bite/year; 19% ≥5 bites/year) are consistent with high exposure levels documented among forestry, nature-management and hunting cohorts and exceed typical general-population seroprevalence or lifetime-ever bitten estimates previously reported (33–35]. This exposure profile provides an epidemiological context for interpreting the prevalence and seroprevalence data presented.

As reported by participants, ticks are mostly encountered in a natural environment (woods or meadows), rather than more anthropized settings such as cultivated fields and urban/peri-urban parks, similar to what was highlighted by Jore and colleagues in Scandinavia [25] or Mathews-Martin and colleagues in France [36]. However, with changes at the human-wildlife interface driving wild animals closer or even into urban settlements, ticks are entering urbanized areas and parks with increasing abundance [21].

Although the use of repellent sprays remains relatively uncommon, behaviours such as conducting body checks upon returning home to promptly remove ticks, and wearing long protective clothing are more widely adopted; this result is similar to that obtained by Blasko-Markic and Socan within a group of TBE patients [24]. These preventive practices are particularly prevalent among individuals who spend more time outdoors, suggesting an increased awareness and sensitivity to the risks associated with tick exposure. By more consistently checking themselves for ticks once home, higher exposed individuals tend also to report a higher tick-finding rate throughout the year. In a study in Poland, researchers highlighted that socioeconomic factors influence the practising of tick-borne diseases prophylactic behaviours [37].

Our results suggest an association between higher relative exposure intensity—including occupational exposure and frequent off-trail activities such as hunting—and higher prevalence and seroprevalence within a uniformly high-risk population. The prevalence and seroprevalence values detected in this study are substantially higher than those reported in general population surveys. Yet, studies employing identical or comparable diagnostic methodologies in similarly exposed populations report equivalent or higher prevalence values, supporting the widespread validity of our findings; the prevalence and seroprevalence values in this study align with expectations for a high-exposure sentinel cohort. Gabrielli et al. [35] detected *B. microti* and *B. divergens* seroprevalence of 32% and 12% respectively in Italian foresters and hunters using IFAT assays comparable to ours (our study: 13.9% and 22.2%). Panczuk et al. [14] documented *B. burgdorferi* IgG prevalence of 63.3% in Polish hunters, substantially higher than our 1.4%, potentially reflecting geographic variation in *Borrelia* species circulation or exposure intensity differences. Multiple Italian studies (36–38] have reported *B. burgdorferi* seroprevalence ranging from 7% to 23% in occupationally exposed populations using validated serological methods. This apparent discrepancy reflects fundamental differences in study population exposure profiles rather than methodological artifacts or unusual geographic variation in pathogen circulation [18, 38–40], which is also supported by the difference in prevalence along the exposure gradient that we detected in the current study.

Conversely, studies reporting substantially lower prevalence typically involve: (1) general population samples with sporadic or low-frequency tick exposure (exposure stratification is essential in tick-borne disease epidemiology); (2) different detection methods with varying sensitivity and specificity profiles, and aims; (3) geographic regions with lower tick abundance or pathogen circulation; or (4) clinical case series representing only a small fraction of infections (the ones progressing to symptomatic disease). The selection of diagnostic tests should be guided by the specific objectives of the study, as variability in test performance characteristics (e.g., sensitivity, specificity, and cut-off thresholds) likely contributes to discrepancies among diagnostic methods and to the occurrence of false-positive results. While such limitations may be acceptable in research and surveillance contexts—where rapid, high-throughput screening is often prioritized—more stringent analytical approaches are warranted for individual-level diagnosis. In these settings, confirmatory testing, refined interpretation, and/or the application of tailored cut-off values should be implemented to ensure diagnostic accuracy.

Four participants reported a previous history of symptoms compatible with tick-borne diseases, including one confirmed case of Lyme borreliosis. However, all participants were asymptomatic at the time of sampling, despite the high level of pathogen exposure, highlighting an important epidemiological principle also observed in wildlife reservoirs: infection prevalence does not necessarily correspond to disease incidence.This dissociation has profound implications for risk assessment, clinical decision-making, and public health surveillance strategies. Specifically, seroprevalence data from high-exposure populations should not be directly extrapolated to predict clinical disease burden in the general population, nor should high seroprevalence in sentinel cohorts be dismissed as methodological artifact without considering exposure context and diagnostic method concordance with published literature. Clinical cases of tick borne diseases are indeed rarely reported in Italy ([41–43]; https://www.epicentro.iss.it/zecche/FebbreBottonosa2001-2015, accessed on 05/10/2024; https://www.ecdc.europa.eu/en/publications-data/notification-rates-locally-acquired-tick-borne-encephalitis-cases-reported-2023, accessed on 05/10/2024). The development of clinical symptoms is substantially rarer than the occurrence of infection, which may obscure underlying epidemiological patterns in studies focusing exclusively on clinical cases.

Being all TBEV seropositive individuals vaccinated against the virus, our data remark that TBEV possibly has not yet expanded to the northwest of Italy, as the absence of reported human cases suggests (Calcagno, personal communication). A single serological study carried out in 2007 in the same region reported a seroprevalence for TBEV of 5.7% [44], with no evaluation of cross-reactivity for other flaviviruses or vaccination.

## Conclusions

By characterizing pathogen circulation across a gradient of exposure intensity, this study provides insights into transmission dynamics at the wildlife-human interface and establishes baseline prevalence data for sentinel populations in Northwestern Italy. Indeed, the rising exposure to tick bites impacts not only those engaged in professional outdoor activities, but also individuals involved in outdoor leisure activities. As demonstrated in wildlife hosts, microorganisms may frequently circulate at high prevalence without causing symptomatic disease in most infected individuals. Comprehensive surveillance of pathogens prevalence and circulation in sentinel human populations is essential for accurately assessing the risks posed by natural environments. Additionally, this information is fundamental in guiding the therapeutical approach towards these diseases [45]. Our data may prompt several interventions:

1. Educational activities on the general population on the risks of TBZ and how to avoid them;
2. Educational activities on healthcare workers on the diseases associated with TBZ;
3. Collaborative surveillance and research studies involving veterinarians, physicians and public health professionals;
4. PCR screening of blood donors engaged in outdoor activities for leisure or work;
5. Molecular surveillance in patients with fever of unknown origin through next-generation sequencing for identifying the clinical burden of TBZ and the circulation of unexpected pathogens.

While some of these activities are already ongoing, this may be a call to take action in this field.

## Supplementary Information

Below is the link to the electronic supplementary material.

Supplementary Material 1

## Data Availability

The datasets used and/or analysed during the current study are available from the corresponding author on reasonable request. The DNA sequences generated during the current study are available in the GenBank repository, accession numbers from PQ657427 to PQ657433 and from PQ684452 to PQ684460.

## Abbreviations

C.I.: Confidence Interval
ELISA: Enzyme-Linked Immunosorbent Assay
IFAT: Immunofluorescence Antibody Test
PCR: Polimerase Chain Reaction
SFG: Spotted Fever Group
TBEV: Tick-Borne Encephalitis Virus
TBZ: Tick-Borne Zoonoses
