# Supplementary material for: “The clock is ticking”: high prevalence of tick-borne zoonoses in healthy adults with varying tick-bite exposure in Northwestern Italy
Source: BMC Infect Dis. 2026 Jul 28;26:1878. doi: 10.1186/s12879-026-14094-9 (PMC13700455; doi:10.1186/s12879-026-14094-9)
Supplement: Supplementary file 1 [file 12879_2026_14094_MOESM1_ESM.docx]

**Supplementary Table 1.** Translated version of the survey submitted to the participants to the study. The original survey was submitted in Italian.

| **Survey** | | | | | | | | | | | | | | | | | | | | | | |
| --- | --- | --- | --- | --- | --- | --- | --- | --- | --- | --- | --- | --- | --- | --- | --- | --- | --- | --- | --- | --- | --- | --- |
| *Demographic data* | | | | | | | | | | | | | | | | | | | | | | |
| Gender: | | | | | | | | | | | | | | | | | | | | | | |
| Age | <20 | | 20-30 | | | | | 31-40 | | | | | 41-50 | | | 51-60 | | | | | >60 | |
| Municipality of residence | | | | | | | | | | | | | | | | | | | | | | |
| *Exposure to tick bite* | | | | | | | | | | | | | | | | | | | | | | |
| Employment: | | | | | | | | | | | | | | | | | | | | | | |
| Do you engage in any of the following activities that expose you to tick bites: | | | | | | | | | | | | | | | | | | | | | | |
| □      Access to a farm with livestock  □      House near the woods  □      Garden with wildlife access  □      Leisure outdoor activity: hunt  □      Leisure outdoor activity: fishing  □      Leisure outdoor activity: camping, trekking, cycling  □      Leisure outdoor activity: mushroom/other products picking | | | | | | | | | | | | | | | | | | | | | | |
| Weekly frequency: | | | | <1 | | 1 | | | 2 | | | 3 | | | 4 | | 5 | | | 6 | | 7 |
| Mean of daily hours spent outdoor: | | | | | | | | | | <10 | | | | | | | | >10 | | | | |
| Do you have any medical conditions? | | | | | | | | | | | | | | | | | | | | | | |
| Long clothing while outdoor | | | | | | | Always | | | | | | | Sometimes | | | | | Never | | | |
| Use of spray repellents while outdoor | | | | | | | Always | | | | | | | Sometimes | | | | | Never | | | |
| Tick check once back home | | | | | | | Always | | | | | | | Sometimes | | | | | Never | | | |
| *Ticks bite* | | | | | | | | | | | | | | | | | | | | | | |
| Annual frequency: | | | | | | | | | | | | | | | | | | | | | | |
| Region: | | | | | | | | | | | | | | | | | | | | | | |
| Environment: | | Wood | | | Meadow | | | | | | Cultivated field | | | | | | Urban/periurban park | | | | | |
| Symptoms after tick bite: | | | | | | | | | | | | | | | | | | | | | | |
| Further comments: | | | | | | | | | | | | | | | | | | | | | | |

**Supplementary Table 2.** Commercial IFAT or ELISA kit implemented to detect IgG antibodies against the pathogens targeted in the study.

| Target | Product implemented | Cross-reactivity detected |
| --- | --- | --- |
| Determination by Indirect Fluorescent Antibody Test (IFAT) | | |
| *B. divergens* | Babesia divergens IgG IFA kit, DVG-120  Fuller Laboratories, Fullerton, California, USA | *Plasmodium* spp. and *Babesia* sp. |
| *B. microti* | Babesia microti IgG IFA Kit, BMG-120  Fuller Laboratories, Fullerton, California, USA | No |
| *A. phagocytophilum* | HGE IgG IFA kit, EEG-120  Fuller Laboratories, Fullerton, California, USA | *A. platys* |
| Determination by inmunoenzimatic essay (ELISA) | | |
| *Rickettsia conorii* | Rickettsia conorii ELISA IgG IgM, VL027  Vircell, Granada, Spain | Spotted Fever Group |
| TBE virus | TBE/FSME IgG ELISA, DETICG0440  Demeditec, Kiel, Germany | Flavivirus |
| *B. burgdorferi* s.l. | BORRELIA IgG (LYME DISEASE) EIA, AVO ESR121G  Institut Virion\Serion GmbH, Wurzburg, Germany | *Leptospira, Treponema pallidum,* Rheumatoid Factor |

**Supplementary Table 3.** PCR protocols implemented in the study. We present the primers and the publication they were taken from and the thermic profile and reaction mixture adapted to the current study.

| Pathogen | Primers (5’ – 3’) | Thermic profile | Reaction mixture  (25 ul) | Reference |
| --- | --- | --- | --- | --- |
| Anaplasmataceae | *16S rRNA*  PER1: TTTATCGCTATTAGATGAGCCTATG  PER2:  CTCTACACTAGGAATTCCGCTAT | 95°C - 15’  40 cycles:  94°C - 1”  52.4°C - 45”  72°C - 1”  72°C - 10’ | HotStarTaq DNA Polymerase: 2.5 U (Qiagen, Hilden, Germany)  Primers: 25 pmol  MgCl2: 0.5 mM  DNA: 1 μl  dNTPs mix: 0.2 mM (Sigma-Aldrich, St. Louis, MO, USA)  PCR buffer: 10X | (1, 2) |
| *B. burgdorferi* s.l. | *spacer region between 5S and 23S rRNA genes*  23SN1: ACCATAGACTCTTATTACTTTGAC  23SC1:  TAAGCTGACTAATACTAATTACCC | 95°C - 15’  Touch down:  94°C - 20”  60°C - 30” (-1°C every cycle)  72°C - 30”  25 cycles:  94°C - 20”  55°C - 30”  72°C - 30”  72°C - 10’ | HotStarTaq DNA Polymerase: 2.5 U (Qiagen, Hilden, Germany)  Primers: 20 pmol  DNA: 2.5 μl  dNTPs mix: 0.2 mM (Sigma-Aldrich, St. Louis, MO, USA)  PCR buffer: 10X | (3) |
| SFG Rickettsia | *surface protein rOmpA*  190-70:  ATGGCGAATATTTCTCCAAAA  90-701:  GTTCCGTTAATGGCAGCATCT | 95°C - 15’  40 cycles:  94°C - 30”  55°C - 30”  72°C - 1’  72°C - 10’ | HotStarTaq DNA Polymerase: 2.5 U (Qiagen, Hilden, Germany)  Primers: 7 pmol  DNA: 5 μl  dNTPs mix: 0.2 mM (Sigma-Aldrich, St. Louis, MO, USA)  PCR buffer: 10X | (4) |

1.	Goodman JL, Nelson C, Vitale B, Madigan JE, Dumler JS, Kurtti TJ, Munderloh UG. 1996. Direct cultivation of the causative agent of human granulocytic ehrlichiosis. N Engl J Med 334:209–215.

2.	Battisti E, Zanet S, Khalili S, Trisciuoglio A, Hertel B, Ferroglio E. 2020. Molecular Survey on Vector-Borne Pathogens in Alpine Wild Carnivorans. Front Vet Sci 7:1–9.

3.	Rijpkema SG, Molkenboer MJ, Schouls LM, Jongejan F, Schellekens JF. 1995. Simultaneous detection and genotyping of three genomic groups of Borrelia burgdorferi sensu lato in Dutch Ixodes ricinus ticks by characterization of the amplified intergenic spacer region between 5S and 23S rRNA genes. J Clin Microbiol 33:3091–3095.

4.	Roux V, Fournier P-E, Raoult D. 1996. Differentiation of spotted fever group rickettsiae by sequencing and analysis of restriction fragment length polymorphism of PCR-amplified DNA of the gene encoding the protein rOmpA. J Clin Microbiol 34:2058–2065.

**Supplementary Table 4.** Prevalence and 95% C.I. intervals for each pathogen and stratification group.

| **Stratification** | **Pathogen** | **Prevalence** | **CIlower** | **CIupper** |
| --- | --- | --- | --- | --- |
| **PCR** | | | | |
| Higher exposure | *A. phagocytophilum* | 9.52 | 5.26 | 16.65 |
| Lower exposure | *A. phagocytophilum* | 0.00 | 0.00 | 10.15 |
| Higher exposure | *B. burgdorferi* | 9.52 | 5.26 | 16.65 |
| Lower exposure | *B. burgdorferi* | 8.82 | 3.05 | 22.96 |
| Higher exposure | *Rickettsia* SFG | 9.52 | 5.26 | 16.65 |
| Lower exposure | *Rickettsia* SFG | 8.82 | 3.05 | 22.96 |
| **Serology** | | | | |
| Higher exposure | *B. divergens* | 25.71 | 18.32 | 34.83 |
| Lower exposure | *B. divergens* | 12.82 | 5.60 | 26.71 |
| Higher exposure | *B. microti-like* | 15.24 | 9.60 | 23.33 |
| Lower exposure | *B. microti-like* | 10.26 | 4.06 | 23.58 |
| Higher exposure | *A. phagocytophilum* | 15.24 | 9.60 | 23.33 |
| Lower exposure | *A. phagocytophilum* | 5.13 | 1.42 | 16.89 |
| Higher exposure | *B. burgdorferi* | 5.00 | 2.15 | 11.18 |
| Lower exposure | *B. burgdorferi* | 2.56 | 0.45 | 13.18 |
| Higher exposure | *Rickettsia* SFG | 19.30 | 11.13 | 31.34 |
| Lower exposure | *Rickettsia* SFG | 2.94 | 0.52 | 14.92 |
| Higher exposure | TBE | 3.85 | 1.51 | 9.47 |
| Lower exposure | TBE | 0.00 | 0.00 | 8.97 |
